# Viability of acellular biologic graft for nipple-areolar complex reconstruction in a non-human primate model

**DOI:** 10.1038/s41598-021-94155-y

**Published:** 2021-07-23

**Authors:** Vincent C. Caronna, Allison F. Rosenberg, David M. Graham, William M. Heim, Brooke F. Grasperge, Scott K. Sullivan, Abigail E. Chaffin, Bruce A. Bunnell, Nicholas C. Pashos

**Affiliations:** 1 BioAesthetics Corporation, Durham, NC USA; 2 Division of Veterinary Medicine, Tulane National Primate Research Center, Covington, LA USA; 3 Center for Restorative Breast Surgery, New Orleans, LA USA; 4 School of Medicine, Department of Surgery, Tulane University, New Orleans, LA USA; 5 School of Medicine, Center for Stem Cell Research and Regenerative Medicine, Tulane University, New Orleans, LA USA; 6 School of Medicine, Department of Pharmacology, Tulane University, New Orleans, LA USA

**Keywords:** Regenerative medicine, Tissue engineering

## Abstract

Many of the > 3.5 million breast cancer survivors in the US have undergone breast reconstruction following mastectomy. Patients report that nipple-areolar complex (NAC) reconstruction is psychologically important, yet current reconstruction techniques commonly result in inadequate shape, symmetry, and nipple projection. Our team has developed an allogeneic acellular graft for NAC reconstruction (dcl-NAC) designed to be easy to engraft, lasting, and aesthetically pleasing. Here, dcl-NAC safety and host-mediated re-cellularization was assessed in a 6-week study in rhesus macaque non-human primates (NHPs). Human-derived dcl-NACs (n = 30) were engrafted on the dorsum of two adult male NHPs with each animal’s own nipples as controls (n = 4). Weight, complete blood counts, and metabolites were collected weekly. Grafts were removed at weeks 1, 3, or 6 post-engraftment for histology. The primary analysis evaluated health, re-epithelialization, and re-vascularization. Secondary analysis evaluated re-innervation. Weight, complete blood counts, and metabolites remained mostly within normal ranges. A new epidermal layer was observed to completely cover the dcl-NAC surface at week 6 (13–100% coverage, median 93.3%) with new vasculature comparable to controls at week 3 (p = 0.10). Nerves were identified in 75% of dcl-NACs (n = 9/12) at week 6. These data suggest that dcl-NAC is safe and supports host-mediated re-cellularization.

## Introduction

Patients who have had mastectomies due to breast cancer indicate that nipple-areolar complex (NAC) preservation or reconstruction is of vital importance to their self-esteem, body image, and quality of life^1–5^. Patients report that NAC reconstruction decreases feelings of impairment and mutilation following mastectomy, and provides a sense of completeness and closure to the cancer experience^1, 2, 6–10^. Nipple-sparing mastectomies (NSM) leave the nipple and areola intact, but clear cancer margins must exist in order to be eligible for NSM; NSM-ineligible patients can elect to not undergo NAC reconstruction or choose between NAC reconstruction methods^4, 11, 12^.

Current NAC replacement and reconstruction approaches, including prostheses, tattoos, and various surgical methods, can produce NACs that are non-living, non-permanent, lack physical depth, are asymmetric, or fail to maintain a nipple projection, leading to revision surgeries that further burden patients^5, 10, 13–18^. Patient satisfaction varies across similar surgical reconstruction techniques, possibly because of differences between the skill and expertise of individual surgeons^16, 18–23^. Tissue engineering approaches have been pioneered, such as subdermal NAC implants and fillers, and complications include sinking of the newly formed nipple protrusion^14, 18, 24–28^. Decellularized skin has been used previously to form an areola^29^. Reported complication rates for nipple reconstruction vary widely, as detailed in an extensive review, as 46.9%, 7.9%, and 5.3% after graft, local flap, and flaps with autologous graft/alloplastic/allograft augmentation, respectively^30^. The majority of complications were attributed to diminution in donor-site sensation (31.3%); pain, irritation, or loss of sensation in the nipple (7.3%); and nipple necrosis (up to 6.3%). Overall, nipple projection loss was found to be 40–75%^30^.

To address the limitations of current approaches, we hypothesized that donor NACs could be decellularized to generate dcl-NAC, an off-the shelf, acellular biologic graft that would provide an optimal scaffold for regeneration of both the nipple and areola, and maintain a nipple and areola for the lifetime of the patient. The dcl-NAC is the first decellularized NAC for NAC reconstruction.

Decellularization removes donor cellular and genetic material while retaining much of the tissue’s endogenous structure and biochemical and biomechanical features^31–34^. Acellular grafts such as dcl-NAC, unlike intact tissue grafts, pose minimal risk of immune rejection and do not require an immediate blood supply to sustain them^33, 35^. Acellular dermal matrices derived from deceased donors have been used commercially in various types of reconstructive surgeries for almost 30 years, and studies have shown that host cells repopulated these scaffolds and formed new blood vessels that sustain the tissue^31, 32, 34, 36, 37^. In prior studies we demonstrated that our decellularization method successfully preserves the extracellular matrix’s (ECM) gross architecture, micro-structures, and > 150 different peptides, providing a non-immunogenic cell-free graft^33–35, 38^. Currently, this level of complexity cannot be recreated synthetically^15, 39, 40^. We also demonstrated in two in vivo feasibility studies that, after subcutaneous implantation in mice and onlay engraftment on a non-human primate (NHP), NHP-derived dcl-NACs re-epithelialize and re-vascularize^33^.

Presented herein is a six-week study in which two NHPs received onlay-engrafted human-derived dcl-NACs. Primary outcomes were animal health and host-mediated re-epithelialization and re-vascularization; a secondary outcome was re-innervation. We demonstrate that dcl-NAC is a safe and viable option, supporting host-mediated re-epithelialization and re-vascularization at week 6, as well as re-innervation, which supports dcl-NAC’s ability to regenerate a living NAC.

## Materials and methods

### NAC tissue recovery, decellularization, and sizing

**Figure 1:**
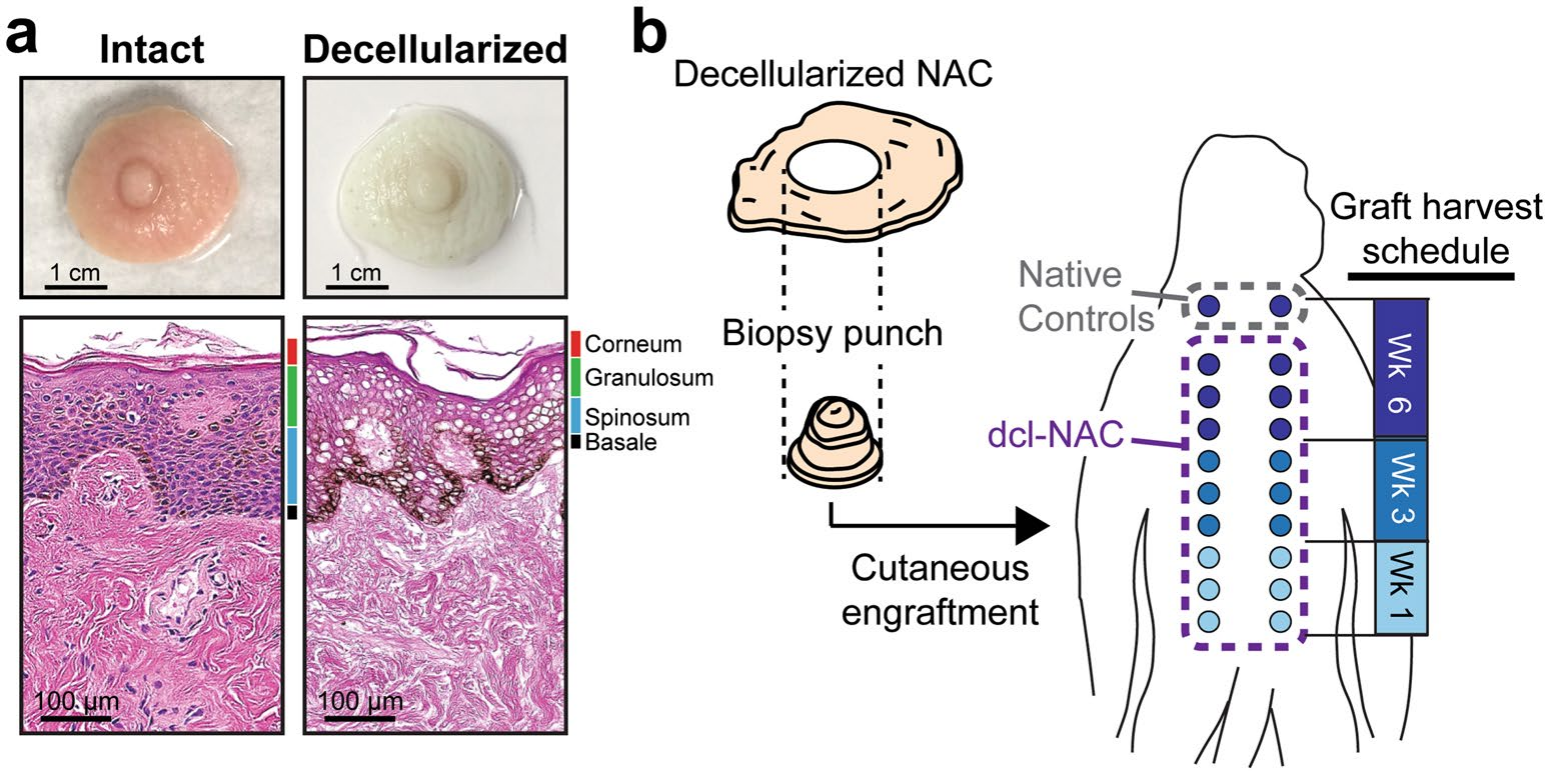
Intact donor NAC vs. decellularized dcl-NAC, and overview of in vivo NHP engraftment study. (**a**) Gross images and H&E sections show an intact donor NAC (left panels) and a decellularized dcl-NAC (right panels). Note that dcl-NAC retains ECM structure and defined dermal layers, and lacks cellular components like DNA (purple staining in lower panels, DNA; brown coloration in strata basale, endogenous melanin). (**b**) Experimental overview: dcl-NACs were biopsy punched to a standard size and engrafted along the dorsal midline of rhesus macaque hosts with the animal’s own two intact, non-decellularized, native nipples as surgical controls. Grafts were harvested at weeks 1, 3, and 6. This was drawn by Vincent Caronna.

Human NACs (Fig. 1a) were recovered from deceased donors by organizations accredited by the American Association of Tissue Banks (AATB); consent is obtained by these organizations from the donor via a Document of Gift (i.e., registered organ donor or consent prior to death) or from next of kin via a Document of Authorization; no tissues were obtained from prisoners. All donor tissue was handled with care and identification numbers that anonymize name, age, gender, and demographic were assigned to all donor tissues and used strictly for tissue processing and tracking purposes. Decellularization was performed as previously described (Fig. 1a)^35^. Dcl-NAC areola size was standardized by biopsy punch to 1.2 cm in diameter (Fig. 1b). This size was chosen to accommodate an n of 6 dcl-NACs per timepoint given the dorsal surface limitations of the NHPs.

### Study design

#### Surgical engraftment and harvest of grafts

Surgical approaches were performed by a plastic and reconstructive surgeon or veterinarian according to the NIH Guide for Care and Use of Laboratory Animals and a protocol approved by the Institutional Animal Care and Use Committee of the Tulane National Primate Research Center (TNPRC, P0337). The Indian rhesus macaque (*Macaca mulatta*) hosts in these studies were two adult males, five to six years of age, selected because females are considered a scarce resource, and because males have more dorsal area compared to females, allowing for a larger engraftment area. Prior to day 0 of these studies, the animals underwent a physical examination, including weight observation, complete blood count (CBC), and serum blood chemistry screen, and were determined to be healthy prior to study admission.

An onlay engraftment method was used for all grafts, and performed as described previously with the following modifications (Fig. 1b)^33^. Surgical scissors were used to excise approximately a circular wound bed of 1.2 cm diameter along the NHP’s dorsal midline. Dcl-NACs and each animal’s own two intact, non-decellularized, native nipples, which served as surgical controls, were engrafted on top of the wound beds (cutaneously) and sutured in place for a total of 20 grafts (18 dcl-NACs and 2 controls) for NHP1 and 14 grafts (12 dcl-NACs and 2 controls) for NHP2. Sutured grafts were covered with topical ointment, non-adherent gauze, and several layers of plain gauze. The NHPs were wrapped with self-adhesive bandage to cover the engraftment area and placed in a onesie and a jacket to prevent manipulation of grafts. Animals did not receive any oral or intravenous antibiotic. Bandage changes occurred weekly during which grafts were evaluated for signs of inflammation including edema and redness, and for signs of adverse events such as necrosis.

Grafts were excised *en bloc* at specified timepoints (Fig. 1b) under sedation at weeks 1 (NHP1, n = 6), 3 (NHP1, n = 6; NHP2, n = 6), or 6 (NHP1, n = 6 with 2 controls; NHP2, n = 6 with 2 controls). The excised grafts were immediately fixed in 10% neutral buffered formalin. Following each excision the skin was sutured closed by the veterinarian. Preliminary data from NHP1 suggested that week 1 was a less-relevant timepoint for recellularization, and as such the week 1 timepoint was excluded for NHP2 to reduce surgical burden. The surgery-based portion of the study occurred over a six-week period after which the animals were observed under this protocol for one to two additional weeks before release to a general colony.

#### Animal care

Animal treatment and housing was in accordance with TNPRC standards. A commercially prepared primate diet was provided with supplemental foods such as fruits or treats. Water was provided ad libitum via an automatic watering device. Single housing was necessary to prevent manipulation of the graft sites by other animals, though other animals were housed in the same room to provide visualization of conspecifics. The Study Coordinator or designated technician observed the animal daily for signs of illness or other abnormalities. Any significant abnormal observation, including species-specific behavioral abnormalities, were reported to the Study Director and the Study Veterinarian.

For minor procedures such as blood collection, animals were anesthetized with ketamine hydrochloride (10 mg/kg, intramuscularly [IM]) or tiletamine hydrochloride/zolazepam (Telazol, 5–8 mg/kg IM). As necessary for clinical diagnostic procedures or major surgery, isoflurane gas inhalation anesthesia was used after induction with acepromazine (0.2 mg/kg IM), glycopyrrolate (0.01 mg/kg IM), and ketamine hydrochloride (10 mg/kg IM). Buprenorphine (0.01 mg/kg IM) or sustained release buprenorphine hydrochloride (0.2 mg/kg subcutaneously) and/or sustained release meloxicam (0.6 mg/kg subcutaneously) were used for post-procedural analgesia.

#### Weight, CBC, blood chemistry, and inflammation

During the study, the animals were sedated for weekly assessments during which veterinarians performed physical examinations and obtained weight measurements and blood samples. On physical exam, animals and the grafts were visually assessed for external signs of inflammation (pain, heat, redness, swelling, and loss of function). Weights and peripheral blood samples (~ 5–8 mL) were collected on the animal’s day of surgery (day 0) and weekly until the completion of the study (6 weeks); all samples were taken immediately prior to any surgery to exclude systemic responses in these measurements. Additional data was collected before and after the study period as part of normal monitoring of animal health by the facilities. Blood cell counts were assessed for signs of inflammation, such as neutrophilia, left shift in neutrophils, toxic change, monocytosis, and/or concurrent lymphopenia. CBC analysis was performed using a Sysmex XP-300 hematology analyzer. Blood chemistry analysis was performed using a Beckman AU400 chemistry analyzer for aspartate amino transferase, alanine amino transferase, blood urea nitrogen, creatinine, ratio of blood urea nitrogen/creatinine, glucose, sodium, potassium, chloride, total serum protein, albumin, globulin, and the ratio of albumin/globulin. Animal weights were compared as percent change to the animal’s weight at day 0, and CBC and blood chemistry values were compared to normal value ranges as determined empirically by TNPRC.

#### Histology

All fixed grafts were cut in half along the sagittal axis of the nipple. Samples were processed, paraffin embedded, sectioned (5 µm), and stained with hematoxylin and eosin (H&E) at the histology core facilities at the Tulane Center for Stem Cell Research and Regenerative Medicine or HistoWiz Inc. (Histowiz.com, Brooklyn, New York). H&E-stained slides were submitted to HistoWiz for brightfield slide scanning, and blocs for additional histology. CD31 (PECAM-1; rabbit polyclonal, catalog No. ab28364; ABCAM, 1:100) stained endothelial cell lumens. Ki-67 (K2; catalog No. PA0230; Leica, 1:800) stained proliferating cells. Pan cytokeratin (AE1/AE3; mouse monoclonal, catalog No. NCL-L-AE1/AE3; Leica, 1:100) stained cytokeratins. Vimentin (EPR3776; rabbit monoclonal, catalog No. AB92547; ABCAM, 1:800) stained dermal fibroblasts. Hematoxylin was used as a counter-stain. An anti-rabbit or anti-mouse secondary was used (catalog No. DS9800; Leica, ready to use). Whole slide scanning (40x) was performed on an Aperio AT2 (Leica Biosystems).

### Epithelialization analysis

Re-epithelialization was measured on scans of H&E-stained sections using Aperio ImageScope software (version 12.3.2.8013) [https://www.leicabiosystems.com/digital-pathology/manage/aperio-imagescope/]. The total length of new cellularized epidermis on the exposed surface of the graft was measured and compared to the total length of exposed graft surface (including non-epithelialized and epithelialized surface) to calculate percent re-epithelialization, as previously described^33^.

### Vascular analysis

Randomized regions (n = 4) within each graft were examined at an 11.5 × magnification. CD31 positive blood vessel lumens were outlined in QuPath (version 0.1.2)^41^. Lumen areas were summed within each region then averaged between the 4 regions, graphed as the per-graft average^33^.

### Innervation analysis

H&E-stained sections from grafts resected at week 6 (n = 12) were assessed by a blinded, independent pathologist for the presence of nerves in Aperio ImageScope.

### Statistical analysis and figure generation

Re-epithelialization percentages were logit transformed and raw re-vascularization areas were log transformed. For each data set, two-way ANOVAs were performed with animals and time as factors; only time was found to be a significant factor. Accordingly, re-vascularization and re-epithelialization data for the two NHPs was pooled and analyzed across time with a one-way ANOVA followed by Tukey’s multiple comparisons test. All calculations and graphs were completed using GraphPad Prism version 8. Figures and artwork were made using Adobe Illustrator version 21.1.3.

### Ethics approval

Human NACs were recovered from deceased donors by organizations accredited by the American Association of Tissue Banks (AATB); consent is obtained by these organizations from the donor via a Document of Gift (i.e., registered organ donor or consent prior to death) or from next of kin via a Document of Authorization; no tissues were obtained from prisoners. All donor tissue was handled with care and identification numbers that anonymize name, age, gender, and demographic were assigned to all donor tissues and used strictly for tissue processing and tracking purposes. These animal studies followed ARRIVE reporting guidelines^42^. Surgical approaches were performed according to the NIH Guide for Care and Use of Laboratory Animals and a protocol approved by the Institutional Animal Care and Use Committee of the Tulane National Primate Research Center (TNPRC, P0337).

## Results

### Dcl-NACs demonstrate safety in NHPs

**Figure 2:**
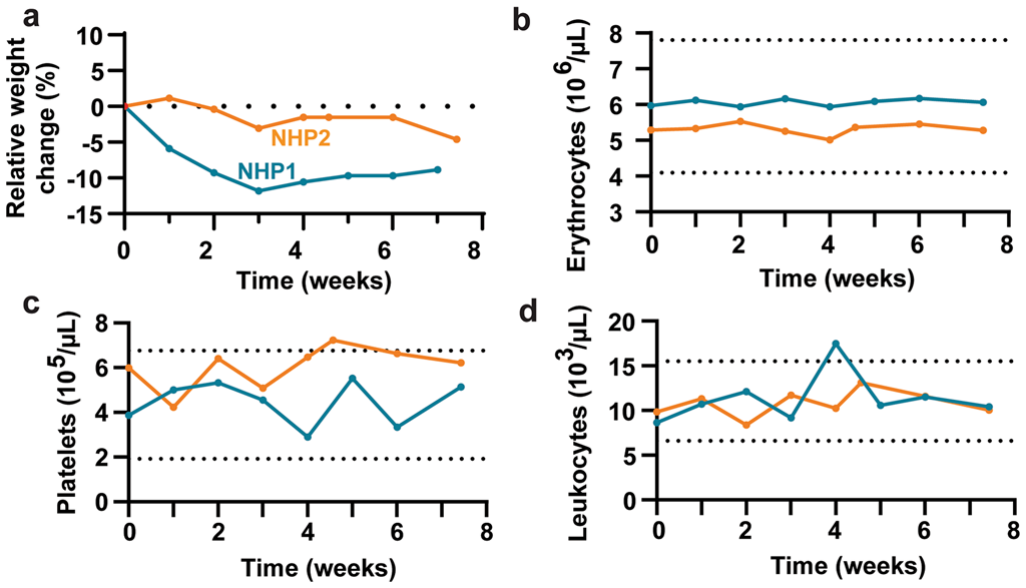
Health status of NHPs after dcl-NAC engraftment. (**a**) Weekly percent change in weight relative to weight at week 0. (**b**–**d**) Weekly erythrocyte, platelet, and leukocyte cell counts. Horizontal dashed lines represent normal range boundaries; blue, NHP1; orange, NHP2.

To determine whether dcl-NACs elicited a systemic immune response or affected NHP health, animal weights were recorded and blood samples were collected for CBC and blood chemistry. Each NHP showed less than a 10% decrease in body weight compared to day 0 except for NHP1 at weeks 3 and 4 (Fig. 2a). Erythrocyte, platelet, and leukocyte counts remained within normal ranges with few exceptions (Fig. 2b–d). Between weeks 4 and 5, NHP2’s platelet count exceeded the upper limits of the normal range, but returned to the upper limit at week 6 (Fig. 2c). NHP1 had an elevated leukocyte count above the normal range at week 4 (Fig. 2d), which occurred in conjunction with mild inflammation at a graft site that day, and resolved by the following week. Blood chemistry results generally remained within normal ranges, showing no unexpected changes (Supplemental Fig. 1). Overall, in the opinion of the veterinarians, both NHPs remained in good health through the course of these studies.

### Dcl-NACs successfully re-cellularize in NHPs

**Figure 3:**
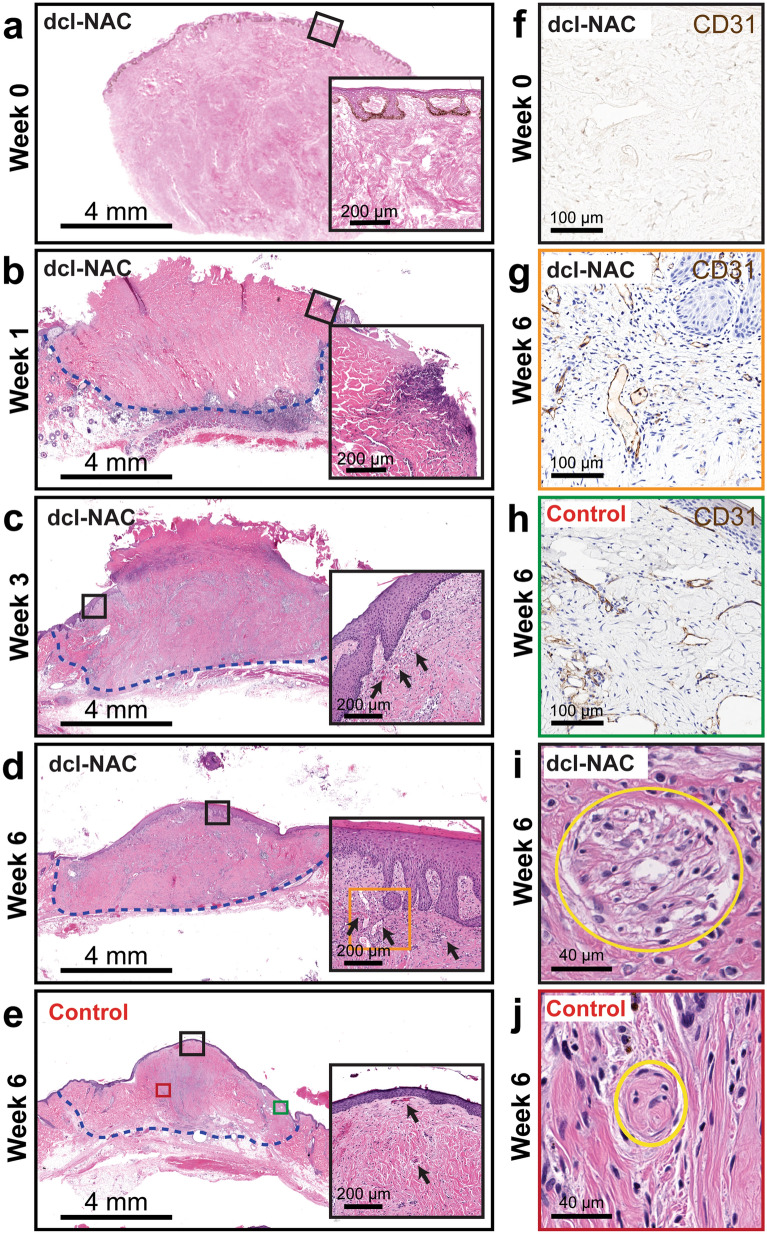
Histological analysis of re-cellularized dcl-NACs and controls. (**a**–**e**) H&E-stained sections from the sagittal midline of dcl-NACs (**a**) prior to engraftment and excised after (**b**) 1 week, (**c**) 3 weeks, (**d**) 6 weeks post-engraftment, and of (**e**) an intact, non-decellularized native NHP control 6 weeks post-engraftment. Black boxes are magnified in insets highlighting surface margin including epidermis, and black arrows point to vasculature; dashed blue line marks graft-host margin. (**f**–**h**) CD31 staining (dark brown) for vasculature in (**f**) a non-engrafted dcl-NAC, (**g**) a dcl-NAC 6-weeks post engraftment (adjacent section magnified from orange box in (**d**)), and (**h**) an intact, non-decellularized native NHP control at week 6 (adjacent section magnified from green box in (**e**)). (**i**, **j**) Yellow circles surround nerves identified in (**i**) a dcl-NAC and in (**j**) an intact, non-decellularized native NHP control (magnified from the red box in **e**) at week 6.

**Figure 4:**
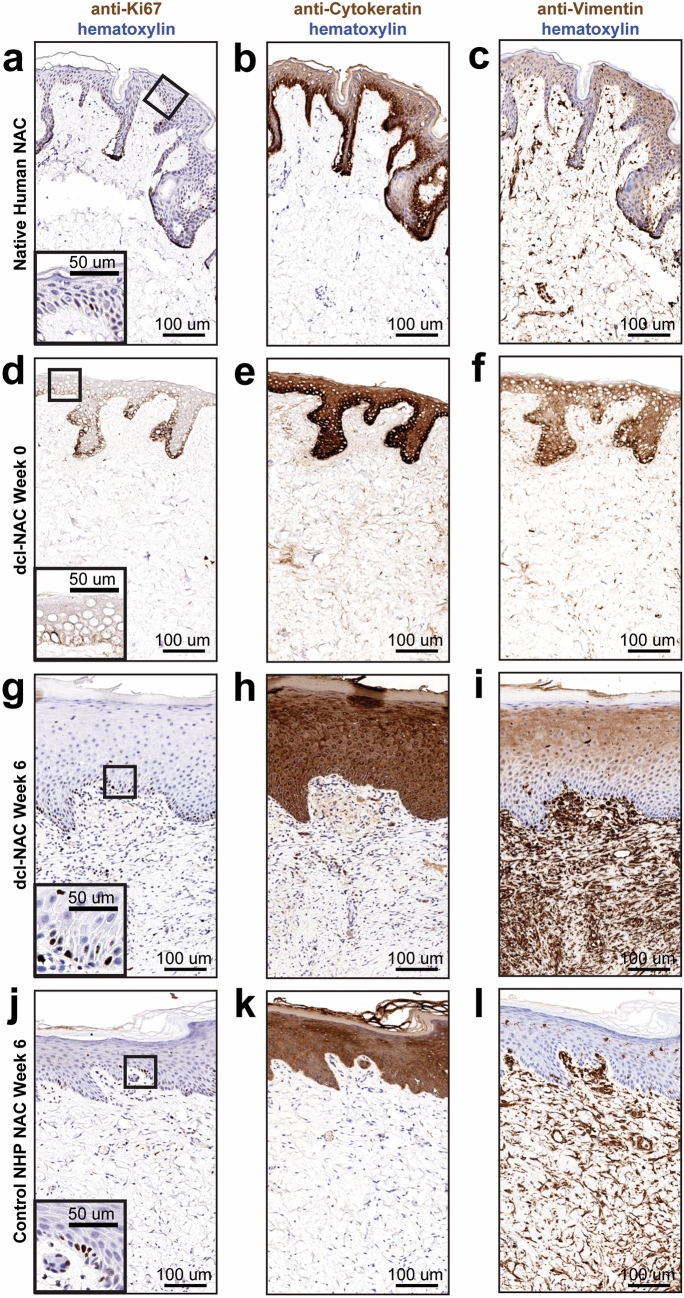
Histological analysis of cell types in re-cellularized dcl-NACs and controls. (**a**, **d**, **g**, **j**) Ki67 (brown) stains proliferative cells. (**b**, **e**, **h**, **k**) Pan-CK (brown) stains keratinized cells. (**c**, **f**, **i**, **l**) Vimentin (brown) stains fibroblasts, and hematoxylin (blue) stains cell nuclei. (**a**–**c**) Intact, non-decellularized human NAC. (**d**–**f**) non-engrafted dcl-NAC. (**g**–**i**) dcl-NAC 6-weeks post-engraftment. (**j**–**l**) Intact, non-decellularized, native NHP NAC 6-weeks post-engraftment.

Histological analysis of resected dcl-NACs focused on determining the degree of re-epithelialization and re-vascularization, which are the benchmarks of long-term graft survivability. Host-mediated re-cellularization was assessed qualitatively. Host cells (purple nuclei) were not present in dcl-NACs prior to engraftment (Fig. 3a) and were observed at week 1 sparsely throughout the dcl-NACs (Fig. 3b) and as far as 4 mm deep, as measured from the base of the graft-host margin. Host nuclei numbers increased over time (Fig. 3b–d). At week 6, dcl-NACs were densely repopulated by host cells, appearing comparable to controls (Fig. 3d vs Fig. 3e). Sections stained with the marker Ki67 for proliferative cells showed a proliferative layer of cells in the stratum basale of a non-decellularized human NAC (Fig. 4a), which was absent following decellularization (Fig. 4d), and restored following epidermal regeneration in a dcl-NAC resected at week 6 (Fig. 4g). As expected, intact, non-decellularized, native NHP controls retained a stratified epidermis and vasculature at the conclusion of the 6-week study (Fig. 3e, inset), and a layer of proliferative cells in the stratum basale (Fig. 4j).

**Figure 5:**
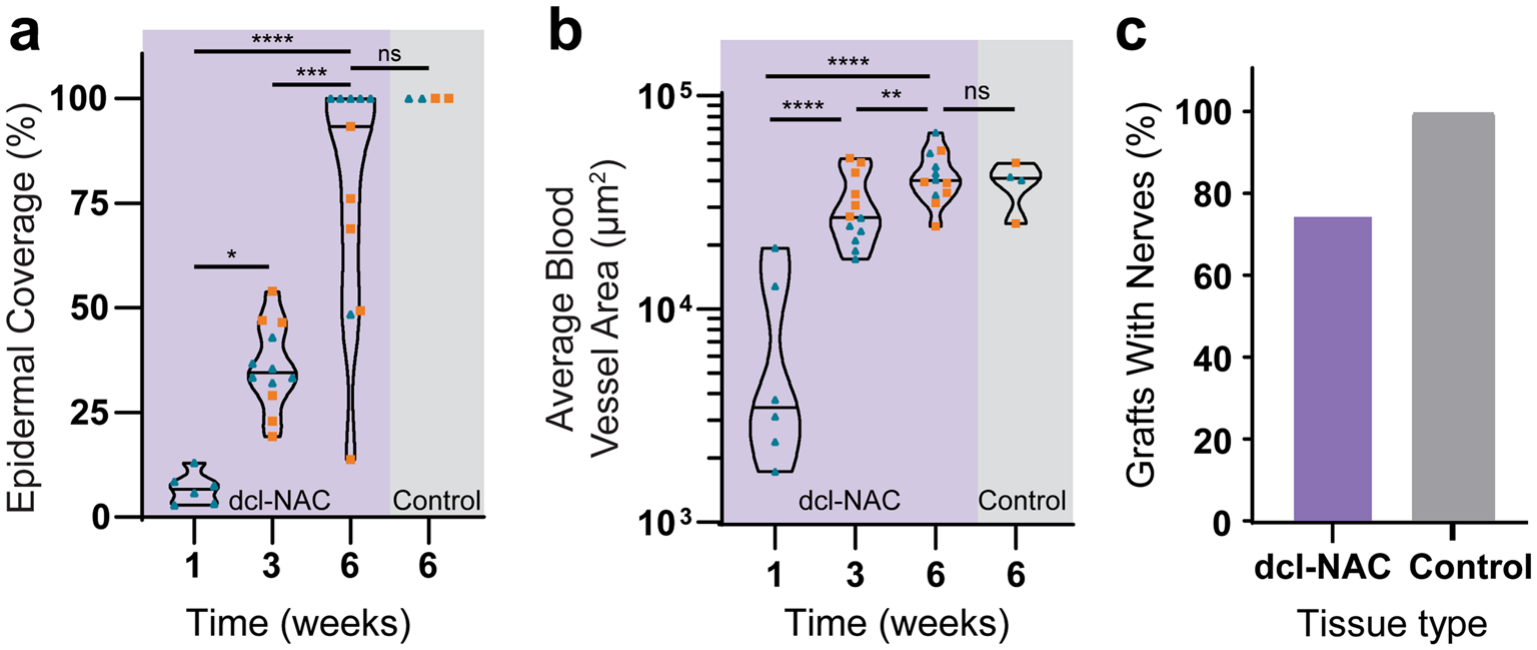
Quantitative analysis of dcl-NAC re-cellularization at study endpoints. (**a**) Percent epidermal coverage of the exposed surface of dcl-NACs and intact, non-decellularized, native NHP controls. (**b**) Average blood vessel area in dcl-NACs and intact, non-decellularized, native NHP controls. Blue triangles, NHP1; orange squares, NHP2. P ≤ 0.05, *; P ≤ 0.01, **; P ≤ 0.001, ***; P ≤ 0.0001, ****; ns, not significant. (**c**) Percent of grafts in which nerves were identified at week 6.

Re-epithelialization of the dcl-NACs was measured as the percentage of the graft’s exposed surface covered by new cellularized epidermis. Prior to engraftment at week 0, dcl-NACs were confirmed to have no living cells in the retained epidermis (0% epidermal coverage; Fig. 3a). At week 1, minimal re-epithelialization was seen in dcl-NACs (median ± SD 6.6% ± 3.8, with a range of 2.8–13.0% epidermal coverage; n = 6 [because week 1 timepoint was not conducted for NHP2]). Significant growth was observed at subsequent timepoints (Fig. 3b compared to Fig. 3c and d, and insets, quantified in Fig. 5a). At week 3, a thick epidermis containing basal cuboidal epithelial cells was visible (median ± SD 34.5% ± 10.2; with a range of 19.2–54.0% epidermal coverage; n = 12). At week 6, epidermal coverage was (median ± SD) 93.3% ± 29.2 (ranging from 13.8 to 100.0%; n = 11 [because the surface of one graft from NHP2 was damaged during processing for histology]). Intact, non-decellularized, native NHP controls maintained epidermal coverage at 100% throughout the study (Fig. 3e, Fig. 4j–l, quantified in Fig. 5a). Stained sections from dcl-NACs at week 6 showed characteristics of viable NACs: proliferating epithelial cells in the basale layer (Fig. 4g, compared with intact, non-decellularized, native human and NHP NAC tissues in Fig. 4a and j, and dcl-NAC at week 0 in Fig. 4d), a keratinized epidermis (Fig. 4h, compared with intact, non-decellularized, native human and NHP NAC tissues in Fig. 4b and k, and dcl-NAC at week 0 in Fig. 4e), and dermal fibroblasts (Fig. 4i, compared with intact, non-decellularized, native human and NHP NAC tissues in Fig. 4c and l, and dcl-NAC at week 0 in Fig. 4f).

Distinct blood vessels, with red blood cells visible inside lumens, were observed throughout all engrafted dcl-NACs and controls. Vasculature was observed and quantified as CD31-positive stained blood vessel lumens (dark brown) lined with hematoxylin-positive cell nuclei (blue) (Fig. 3g, compared with native NHP control Fig. 3h, and non-engrafted dcl-NAC control Fig. 3f which shows minimal CD31 background staining). Vasculature was also identified in H&E-stained sections by endothelial cell morphology (Fig. 3c–e, black arrows point to red blood cells within vasculature). A significant increase in the area occupied by new blood vessels was observed among weeks 1, 3, and 6 in dcl-NACs (mean ± SD = 7188 ± 7209 µm^2^, n = 6 [because week 1 timepoint was not conducted for NHP2]; 30,610 ± 11,559 µm^2^, n = 12; and 42,548 ± 11,726 µm^2^, n = 12, respectively; Fig. 5b). The area occupied by blood vessels at weeks 3 and 6 was not significantly different from intact, non-decellularized, native NHP controls at week 6 (38,894 ± 9816 µm^2^, n = 4; Fig. 5b).

H&E-stained slide sections of grafts resected at week 6 were assessed by an independent, blinded pathologist for the presence of nerves. Nerves (Fig. 3i and j) were identified histologically in 75% of dcl-NACs (n = 9/12) and 100% of intact, non-decellularized, native NHP controls (n = 4/4; Fig. 5c).

## Discussion

Dcl-NAC was developed to provide patients with a lasting, living NAC while providing surgeons with an option that is standardized, off-the-shelf, and reliable. The results from this in vivo study in NHPs demonstrate the viability of this approach given dcl-NAC’s safety profile and ability to support host-mediated re-cellularization.

Animal weights, blood cell counts, and blood chemistry results demonstrate that dcl-NACs are safe and do not cause a potent or adverse immune response in NHP hosts. Overall, veterinarians found animals to be in good health throughout the study and attributed any values outside of normal ranges to surgical manipulation and expected mild inflammation of engraftment/excision sites. Blood chemistries remained within normal ranges, indicating that organ function was unchanged. Leukocyte levels were slightly elevated, which was expected from routine surgical procedures and bandage changes, though mostly remained within normal ranges, demonstrating little to no systemic immune response. Changes in red blood cells were expected, as animals lost blood following each surgery. Study veterinarians attributed fluctuations in weight to expected habit alterations during study procedures, such as reduced appetite following weekly anesthetic events, and considered these weight changes to be normal and not harmful to animal health. This evidence of safety is consistent with the roughly 30 years of safe use of decellularized allografts for various medical applications, including reconstruction of pressure ulcers, burn wounds, surgical wounds, and more^31, 32, 43^.

In addition to providing natural ECM for host cells to grow into, decellularized grafts provide growth factors and other elements that promote re-epithelialization and re-vascularization, which are critical for tissue health and survival^31–34, 44^. Our prior in vitro and in vivo studies using NHP-derived dcl-NACs showed that dcl-NACs are rich in proteins that promote cell migration, adhesion, and differentiation, and support re-epithelialization and re-vascularization^33^. Consistent with our prior in vivo work, the data presented herein demonstrate that, in an NHP model, onlay-engrafted, human-derived dcl-NACs begin to re-epithelialize and re-vascularize within one week of engraftment, suggesting that the dcl-NAC’s endogenous ECM structure and signaling molecules are supportive of host cell migration into the graft. Importantly, these data indicate that migration and regrowth are sustained through 6 weeks post-engraftment, at which point complete re-epithelialization is first observed and re-vascularization is seen at levels comparable to that of intact, non-decellularized, native controls. We did find in some histological sections that the original, decellularized epidermis of some dcl-NACs appears to have partially sloughed off. Our decellularization process generally allows the epidermis of the donor tissue to be retained, however, we attribute this epidermal appearance to either partial loss during processing for decellularization or histology, or to mechanical aggravation during engraftment, bandaging, or removal. Overall, these re-epithelialization and re-cellularization data, combined with the evidence that host cells in the new epidermis have differentiated appropriately into keratinized cells, suggest that engrafted NAC scaffolds are efficiently and effectively integrated into surrounding host tissue. This is consistent with studies of decellularized grafts used in a range of tissue reconstructions, including use of decellularized dermal grafts to form an areola^29, 32, 44–47^, suggesting that dcl-NAC allows in vivo host cell migration into the graft and supports subsequent host cell growth and differentiation.

Limitations of this study were the lack of macroscopic analysis of NAC color and nipple projection, and its short 6 week duration. While nerves were identified within dcl-NACs at 6 weeks post-engraftment, proper assessment of functional nerve recovery and sensation will require evaluation in human patients, and outcomes will likely be heavily case dependent. Future clinical studies of dcl-NACs will investigate wound healing and aspects critical to patient satisfaction such as nipple projection, shape, color, and sensitivity over a 12-month period.

## Conclusion

This study demonstrates that the human-derived dcl-NAC is safe and supports re-cellularization, showing full epithelial coverage, vasculature comparable to intact, non-decellularized, native tissue, and the presence of nerves at 6 weeks post-engraftment. No adverse systemic responses were detected. Our results suggest that the dcl-NAC presents a solution for the regeneration of the NAC for patients who have had a mastectomy.

## Supplementary Information

Supplementary Information.

## Data Availability

The data that support the findings of this study are available from the authors upon reasonable request and with permission of BioAesthetics Corporation. Restrictions apply to the availability of these data, which were used under license for the current study, and are not publicly available.
