# Supplementary material for: Viability of acellular biologic graft for nipple-areolar complex reconstruction in a non-human primate model
Source: Sci Rep. 2021 Jul 23;11:15085. doi: 10.1038/s41598-021-94155-y (PMC8302621; doi:10.1038/s41598-021-94155-y)
Supplement: Supplementary file 1 — Supplementary Information. [file 41598_2021_94155_MOESM1_ESM.pdf]

Viability of Acellular Biologic Graft for Nipple-Areolar Complex Reconstruction in a Non-Human Primate Model

Vincent C. **Caronna**[a^], Allison F. **Rosenberg**, PhD[a^], David M. **Graham**, PhD[a], William M. **Heim**, MS, CTBS[a], Brooke F. **Grasperge**, DVM[b], Scott K. **Sullivan**, MD, FACS[c], Abigail E. **Chaffin**, MD, FACS[d], Bruce A. **Bunnell**, PhD[e], and Nicholas C. **Pashos**, PhD, CTBS[a*]

^Co-first authors

[a]BioAesthetics Corporation, Research Triangle Park, North Carolina, USA.

[b]Tulane National Primate Research Center, Division of Veterinary Medicine, Covington, Louisiana, USA.

[c]Center for Restorative Breast Surgery, New Orleans, Louisiana, USA.

[d]Tulane University, School of Medicine, Department of Surgery, New Orleans, Louisiana, USA.

[e]Tulane University, School of Medicine, Center for Stem Cell Research and Regenerative Medicine, New Orleans, Louisiana, USA; Tulane University, School of Medicine, Department of Pharmacology, New Orleans, Louisiana, USA.

***Corresponding Author:**

Nicholas C. Pashos, PhD.

BioAesthetics Corporation

6 Davis Drive, Suite 828

Research Triangle Park, North Carolina 27709

npashos@bio-aesthetics.com

ORCiD ID: https://orcid.org/0000-0002-3491-8042

Supplemental Figures

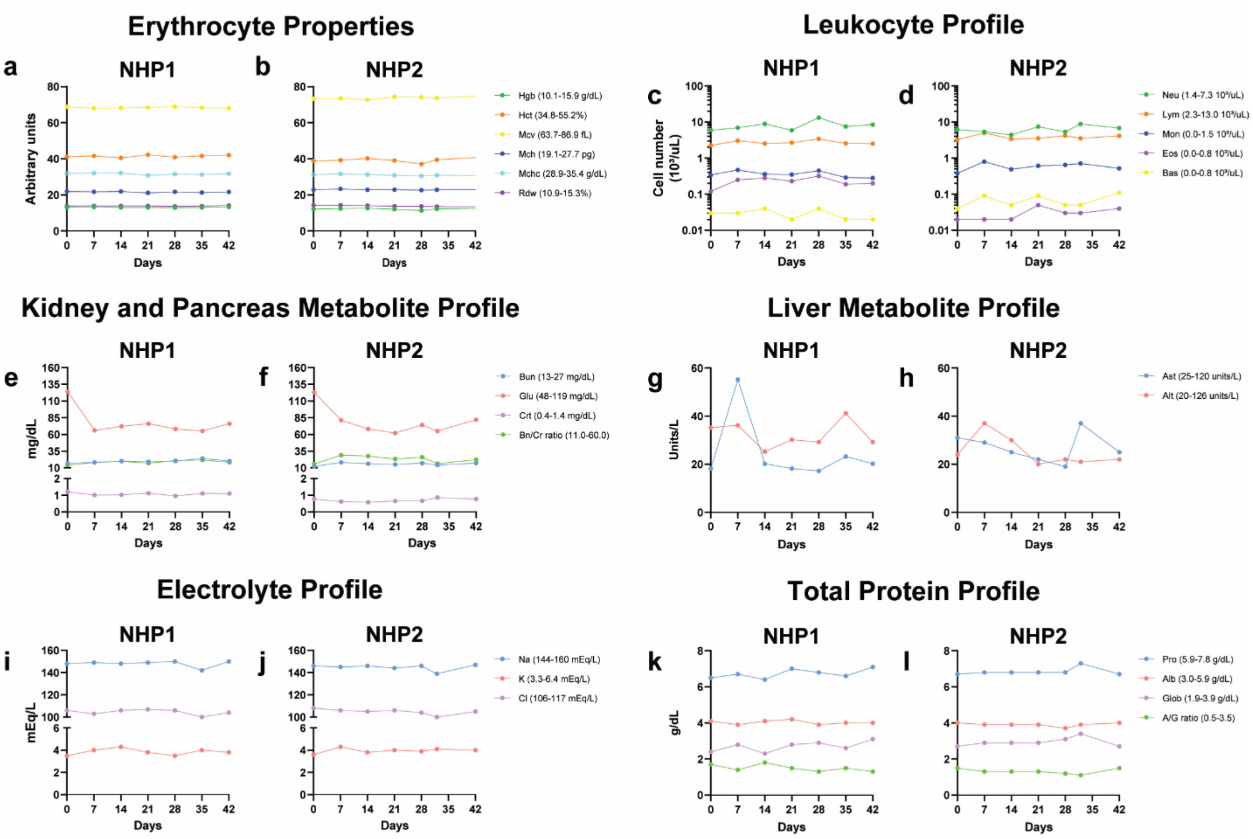

Supplemental Figure 1. Complete blood count and serum blood chemistry analysis. a-b) Erythrocyte properties. Hct, hematocrit; Mcv, mean corpuscular volume; Mch, mean corpuscular hemoglobin; Mchc,mean corpuscular hemoglobin per cell; Rdw, red blood cell distribution width. c-d) Leukocyte profiles. Neu, neutrophil; Lym, lymphocyte; Mon, monocyte; Eos, eosinophil; Bas, basophil. e-f) Kidney and pancreas metabolite profiles. Bun, blood urea nitrogen; Glu, glucose; Crt, creatinine; Bun:Crt is the ratio of blood urea nitrogen to creatinine. g-h) Liver metabolite profiles. Ast, aspartate amino transferase; Alt, alanine amino transferase. i-j) Electrolyte profiles. Na, sodium; K, potassium; Cl, chlorine. k-l) Total protein profiles. Pro, proline; Alb, albumin; Glob, globulin; Alb:Glob is the ratio of albumin to globulin. Normal ranges, determined empirically at TNPRC, are shown in each figure legend. g/dL, grams/deciliter; %, percentage; fL, femtoliter; pg, picogram; 10^3/uL, cell number/microliter; mg/dL, milligrams/deciliter; units/L, units/liter; mEq/L, milliequivalents/liter.
